# PARP-1 regulates inflammasome activity by poly-ADP-ribosylation of NLRP3 and interaction with TXNIP in primary macrophages

**DOI:** 10.1007/s00018-022-04138-z

**Published:** 2022-01-30

**Authors:** Ling-Ya Chiu, Duen-Yi Huang, Wan-Wan Lin

**Affiliations:** 1 Department of Pharmacology, College of Medicine, National Taiwan University, Rm. 1119, 11F., No. 1, Sec. 1, Ren Ai Rd., Zhongzheng Dist., Taipei, 100 Taiwan; 2 Department and Graduate Institute of Pharmacology, National Defense Medical Center, Taipei, Taiwan; 3 Graduate Institute of Medical Sciences, Taipei Medical University, Taipei, Taiwan

**Keywords:** PARP-1, Inflammasome, TXNIP, NLRP3, ROS

## Abstract

Poly(ADP-ribose) polymerase-1 (PARP-1) plays an essential role in DNA repair by catalyzing the polymerization of ADP-ribose unit to target proteins. Several studies have shown that PARP-1 can regulate inflammatory responses in various disease models. The intracellular Nod-like receptor NLRP3 has emerged as the most crucial innate immune receptor because of its broad specificity in mediating immune response to pathogen invasion and danger signals associated with cellular damage. In our study, we found NLRP3 stimuli-induced caspase-1 maturation and IL-1β production were impaired by PARP-1 knockout or PARP-1 inhibition in bone marrow-derived macrophages (BMDM). The step 1 signal of NLRP3 inflammasome activation was not affected by PARP-1 deficiency. Moreover, ATP-induced cytosolic ROS production was lower in *Parp-1*^−/−^ BMDM, resulting in the decreased inflammasome complex assembly. PARP-1 can translocate to cytosol upon ATP stimulation and trigger the PARylation modification on NLRP3, leading to NLRP3 inflammasome assembly. PARP-1 was also a bridge between NLRP3 and thioredoxin-interacting protein (TXNIP) and participated in NLRP3/TXNIP complex formation for inflammasome activation. Overall, PARP-1 positively regulates NLRP3 inflammasome activation via increasing ROS production and interaction with TXNIP and NLRP3, leading to PARylation of NLRP3. Our data demonstrate a novel regulatory mechanism for NLRP3 inflammasome activation by PARP-1. Therefore, PARP-1 can serve as a potential target in the treatment of IL-1β associated inflammatory diseases.

**Supplementary Information:**

The online version contains supplementary material available at 10.1007/s00018-022-04138-z.

## Introduction

PARP-1, which stands for poly(ADP-ribose) polymerase-1, is a multifunctional nuclear enzyme and has key roles in DNA repair, chromatin replication, transcriptional regulation and cell death [1–4]. Upon activation, PARP-1 can use NAD^+^ as the substrate and catalyze the polymerization and attachment of highly negative charged poly(ADP-ribose) (PAR) complex to target proteins, thus regulating protein–protein interaction and chromatin structure. PARP-1 activation plays an essential role in DNA repair under moderate stress; however, in several pathological situations that involve massive DNA damage, extensive activation of PARP-1 would deplete cellular NAD^+^ and its precursor ATP, leading to irreversible cellular energy failure and cell death [4–7]. To date, PARP-1 inhibitors have been approved for the treatment of several types of cancer patients [8], and PARP inhibitors might be an innovative approach to the treatment of inflammatory disorders [9–11] and neurodegenerative diseases [12, 13].

NLRP3 inflammasome, which stands for NACHT domain-, leucine-rich repeat-, and pyrin domain-containing protein 3 (also known as NALP3 or cryopyrin), is a cytosolic multi-protein complex. It can drive inflammatory responses by promoting the maturation of IL-1β and IL-18 in innate immune cells like monocytes and macrophages through two intracellular steps generally [14]. The first step (step 1) is the NF-κB-dependent transcriptional upregulation of pro-IL-1β and NLRP3 triggered by signal 1. Activation of TLR4 by lipopolysaccharide (LPS) is the best known stimulator of signal 1. Signal 2 results in NLRP3 inflammasome complex assembly as step 2, containing the binding of NLRP3 with adaptor protein ASC (apoptosis-associated speck-like protein containing a CARD domain) and recruitment of procaspase-1. The assembled NLRP3 protein complex serves as a platform for procaspase-1 maturation that subsequently mediates the cleavage of pro-IL-1β to mature IL-1β. Signal 2 is provided by a large array of danger signal stimulation, including microbial products, toxins, adenosine triphosphate (ATP), crystalline, and aggregated particles [15, 16]. The detailed mechanism of NLRP3 inflammasome formation triggered by diverse origins and structures of stimuli remains largely unclear. However, previous studies have shown that reactive oxygen species (ROS), potassium efflux, lysosomal rupture, and oxidized mitochondrial DNA releasing from damaged mitochondria are potential activators of the NLRP3 inflammasome assembly [17–22]. In this aspect, thioredoxin-interacting protein (TXNIP) has been demonstrated to mediate NLRP3 inflammasome activation. In basal state, TXNIP associates with thioredoxin (TRX) and inhibits the anti-oxidant activity of TRX. After the stimulation of NLRP3 activators, TNXIP can dissociate from TRX and bind with NLRP3 for activation of inflammasome [18, 23].

In addition to DNA repair, studies over the past decades have begun to reveal the roles of PARP-1 contributing to the regulation of inflammation and host–pathogen interactions [4, 24, 25]. PARP-1 can be simultaneously activated under inflammatory responses and in various disease models. For example, *Parp*-1^−/−^ mice are more resistant to LPS-induced systemic inflammation and streptozotocin-induced diabetes [26]. Moreover, PARP-1 inhibitor olaparib alleviates chronic asthma-associated airway inflammation and remodeling features as well as inhibits NLRP3 inflammasome-mediated IL-1β release [27]. Olaparib administration also can ameliorate the neurological deficits by modulating inflammasome activation in the mouse model of Huntington’s disease [28]. From these studies, PARP-1 is suggested to involve in the activation of NLRP3 inflammasome; however, the molecular mechanisms are not elucidated. Thus, in this study we used genetic and pharmacological approaches to examine the role of PARP-1 in NLRP3 inflammasome activation in LPS-primed macrophages. We found a novel molecular mechanism for NLRP3 inflammasome assembly that is controlled by PARP-1 via PARylation- and ROS-dependent pathways.

## Materials and methods

### Reagents and antibodies

We bought DMEM, FBS, penicillin and streptomycin from Gibco BRL (Grand Island, NY, USA); Opti-MEM, trypsin–EDTA, and Lipofectamine 2000 from Invitrogen (Carlsbad, CA, USA). N-methyl-N'-nitro-N'-nitrosoguanidine (MNNG) was obtained from Chem Service (West Chester, PA, USA); Monosodium urate (MSU) (tlrl-msu) from InvivoGen (San Diego, CA, USA); Imject aluminum gel (Alu) from Thermo Scientific (Rockford, IL, USA). We also purchased specific antibodies of PARP-1, P2X7R, GFP-tag and Myc-tag from Cell Signaling Technology (Danvers, MA, USA). LPS, ATP, protease inhibitor cocktails, and active GST-hPARP-1 protein were from Sigma-Aldrich (St. Louis, MO, USA), and rhGST-NLRP3 was from Abnova (Taipei City, Taiwan). We also purchased polyclonal antibodies specific for horseradish peroxidase (HRP)-conjugated anti-mouse and anti-rabbit, and antibodies directed against mouse caspase-1 p10 (M-20), HA-tag, GST-tag, lamin B and β-tubulin from Santa Cruz Biotechnology (Santa Cruz, CA, USA). Antibody directed against mouse IL-1β and the mouse IL-1β ELISA kit (Cat.: DY401) were from R&D Systems (Minneapolis, MN, USA). Antibodies directed against NLRP3 and ASC were from Adipogen International (San Diego, CA, USA). Antibody against PAR was obtained from BD Pharmingen (San Diego, CA, USA). Antibody against TXNIP was purchased from MBL International Co. (Woburn, MA, USA). Antibody directed against β-actin was from Millipore (Billerica, MA, USA). Cell surface marker CD11b-FITC and Ly6G/6C-PE were obtained from BioLegend (San Diego, CA, USA). 3,4-Dihydro-5[4-(1-piperindinyl) butoxy]-1(2H)-isoquinoline (DPQ), chloromethyl 2′ -7′-dihydrofluorescein diacetate (CM-H2DCFDA), and N-acetyl-L-cysteine (NAC) were obtained from Sigma-Aldrich (St. Louis, MO, USA). MitoSOX™ was purchased from Invitrogen-Molecular Probes (Eugene, OR, USA). CellTiter-Glo® Luminescent kit was purchased from Promega (Madison, WI, USA). Nuclear Extraction Kit was purchased from Cayman (Ann Arbor, MI, USA). The ECL reagent was purchased from Millipore and PerkinElmer (Waltham, MA, USA). We also obtained TriPure Isolation Reagent, FastStart SYBR Green Master, and Genopure Plasmid Maxi Kit from Roche Diagnostics (Indianapolis, IN, USA). The protein concentration was determined by use of a Bio-Rad protein assay (Hercules, CA, USA).

### Mice

*Parp*-KO mice and WT (129/SvImJ) counterparts for *Parp*-KO mice were purchased from Jackson Labs (Bar Harbor, ME, USA) and bred under specific pathogen-free conditions at the Animal Center of National Taiwan University College of Medicine. Nlrp3-KO mice were kindly provided by Dr. Betty A Wu-Hsieh’ Lab. The animal experiments were conducted in accordance with institute regulations after receiving approval from the Ethics Committee of the National Taiwan University College of Medicine (IUCAC No. 20130391).

### Cell culture

Bone marrow-derived macrophages (BMDM) were collected from eight to twelve weeks old WT and *Parp-1*^*−/−*^ mice. The femurs and tibias were flushing with DMEM until bone cavity became white. Cell suspensions were centrifuged at 1000 rpm for 10 min. Then the cells were re-suspended and cultured in 150 mm plastic tissue-culture dishes (Corning, NY, USA) with 20 ml DMEM comprising 10% FBS and 15% L929 fibroblast conditional medium as a source of macrophage colony stimulating factor. The cells were incubated at 37 °C in a humidified atmosphere of 5% CO_2_ and 95% air. After 7 days culture, macrophages were acquired of adherent cells. The preparation of L929 fibroblast conditional medium was collecting the supernatant of 5 × 10^5^/ml L929 fibroblasts cultured for 7 days. L929 and HEK 293 T cells were cultured in complete DMEM.

### *Candida albicans* infection

BMDM were stimulated in OptiMEM serum-free medium at 1 × 10^6^ cells/ml in 6-well plates. Before stimulation, *Candida albicans* was expanded overnight on YPD agar plates (BD Bioscience) at 30 °C. After infection with *C. albicans* for 6 h, BMDM were washed twice with PBS. Experiments were carried out at a multiplicity of infection (MOI) of 10.

### UV irradiation

For cell studies, BMDM were incubated in PBS, then immediately subjected to UVB irradiation. UV21 9w Broadband UVB lamp (Waldmann, Villingen-Schwenningen, Germany), which emitting ultraviolet rays between 280 and 320 nm was used as the light source. BMDM were placed 10 cm below the lamp and irradiated. To achieve accurate UVB dosages, we used the dosimeter VARIOCONTROL (Waldmann, Villingen-Schwenningen, Germany), which was equipped with a specific probe, to calibrate the irradiance intensity periodically.

### ELISA assays of IL-1β

Following incubation with the ligands, the concentrations of mouse IL-1β in the culture medium were determined by use of ELISA in accordance with the manufacturer’s instruction.

### Immunoblot analysis

After stimulation, the medium was aspirated. Cells were rinsed twice with ice-cold PBS, and 25 ~ 100 µl of cell lysis buffer (20 mM Tris–HCl, pH 7.5, 125 mM NaCl, 1% Triton X-100, 1 mM MgCl_2_, 25 mM β-glycerophosphate, 50 mM NaF, 100 µM Na_3_VO4, 1 mM PMSF, 10 µg/ml leupeptin, and 10 µg/ml aprotinin) was then added to each well. After harvesting, cell lysates were sonicated and centrifuged, and equal protein amounts of soluble protein, as determined by the Bradford protein assay, were denatured, subjected to sodium dodecyl sulfate polyacrylamide gel electrophoresis (SDS-PAGE), and transferred to a polyvinylidene difluoride (PVDF) membrane. Nonspecific binding was blocked with TBST (50 mM Tris–HCl, pH 7.5, 150 mM NaCl, and 0.02% Tween 20) containing 5% nonfat milk for 1 h at room temperature. After immunoblotting with the first specific antibody, membranes were washed three times with TBST and incubated with an HRP-conjugated secondary antibody for 1 h. After three washes with TBST, the protein bands were detected with enhanced chemiluminescence detection reagent. Equal amounts of sample protein were applied for electrophoresis and immunoblotting, and β-actin was used as an internal control.

### Immunoprecipitation

To examine protein–protein interaction and protein complex formation, we washed stimulated cells twice with PBS, lysed the cells in 500 µl of RIPA lysis buffer containing 150 mM NaCl, and centrifuged the samples at 14,000 rpm for 15 min at 4 °C. Supernatants were collected, precleared with normal IgG, and incubated with protein A-agarose beads for 30 min. After centrifugation, supernatants were subjected to immunoprecipitation with 0.5 µg of the specific primary antibody for 16 h, and 10 µl of protein A-agarose beads was added before samples were rotated at 4 °C for a further 1 h. Coimmunoprecipitated protein complexes were washed twice with cold RIPA lysis buffer containing 300 mM NaCl and three times with RIPA lysis buffer containing 150 mM NaCl. Beads were added to sample loading buffer and boiled. After centrifugation, the supernatant was subjected to 8–10% SDS-PAGE, followed by Western blotting analysis as described earlier.

### Reverse transcription and real-time quantitative PCR

To measure specific gene expressions, the primer sequences for IL-1β, NLRP3 and β-actin were synthesized (Table 1). Following LPS treatment, the cells were homogenized with 300 μl TriPure Isolation Reagents (Roche Diagnostics), and 2 μg total RNA was reverse transcribed with a RT-PCR kit (Promega, Heidelberg, Germany), in accordance with the manufacturer instructions. The FastStart SYBR Green Master was used to perform the real-time PCRs in 96-well plates, and an ABI Prism 7900 was used to determine the PCR products.

**Table 1:** List of primer sequences used for real-time RT-PCR analysis

Gene	Forward sequence (5′–3′)	Reverse sequence (3′–5′)
*il-1β*	GCTTCAGGCAGGCAGTATCAC	CGACAGCACGAGGCTTTTT
*nlrp3*	AGAGAATGAGGTCCTCTTTACCATGT	AGCCCCGTGCACACAATC
*β-actin*	CGGGGACCTGACTGACTACC	AGGAAGGCTGGAAGAGTGC

### Plasmids and transient transfections

The full length PARP-1 plasmids and Myc-tagged NLRP3 were generated in our laboratory [29]. The GFP-TXNIP was purchased from ADDGENE (Cambridge, MA, USA). The deletion forms of PARP-1 and TXNIP plasmids were also generated in our laboratory. All plasmids were confirmed by DNA sequencing. HEK 293 T cells (5 × 10^5^ cells per well) were transfected with 1 μg of plasmid DNA. After 4–6 h, medium containing the transfection reagents was replaced with complete medium. Twenty-four hours after transfection cells were treated with the reagents indicated in the figure legends, lysed, and then subjected to immunoprecipitation and Western blotting analysis.

### ASC cross-linking assay

The treated primary macrophages were used to perform the ASC oligomerization experiment in accordance with previous research. The cells were resuspended briefly in ASC lysis buffer A, and the lysates were sheared by passing them through a 27-G needle 10 times. The cell lysates were centrifuged at 340 x *g* for 8 min to remove the intact cells and nuclei. The supernatants were added with 1 volume of CHAPS buffer, and the pellets containing the ASC were collected by centrifugation at 2650 x *g* for 8 min. Subsequently, the crude pellets were resuspended in CHAPS buffer containing chemical cross-linked reagent (4 mM disuccinimidyl suberate; Cayman Chemical, Ann Arbor, MI, USA), incubated at room temperature for 30 min.

### Cellular ROS detection

To measure cellular ROS, we used CM-H2DCFDA, which can readily enter cells and be cleaved by esterase to yield DCFH, a polar, non-fluorescent product. ROS in cells promote the oxidation of DCFH to yield the fluorescent product, dichlorofluorescein. After treatment for the indicated time periods, cells were collected and then incubated in PBS containing the reagent CM-H2DCFDA (5 μM) for 30 min at 37 °C. After incubation, cells were washed by PBS twice, trypsinized, re-suspended in 0.5 ml PBS, and immediately submitted to flow analysis using a FACScan flow cytometer. The data based on the FL1 channel were analyzed with the CellQuest program. To measure mitochondrial ROS, we used MitoSOX™, which is a live-cell permeant and is rapidly and selectively targeted to mitochondria. Once in the mitochondria, MitoSOX™ Red reagent is oxidized by superoxide and exhibits red fluorescence (with excitation at 510 nm and emission at 580 nm). After drug treatment for the indicated time periods, cells were collected and then incubated in PBS containing 5 μM MitoSOX™ for 30 min at 37 °C. After incubation, cells were washed with PBS twice, then trypsinized, re-suspended in 0.5 ml PBS, and immediately submitted to flow analysis. Data based on the FL2 channel were analyzed with the CellQuest program.

### Nuclear fractionation

After treatment with LPS and ATP, nuclear extracts were isolated using the Nuclear Extraction Kit according to the manufacturer’s protocol. Briefly, 5 × 10^6^ cells were washed with cold PBS twice. Buffer A (100 μl; 10 mM HEPES, pH 7.9, 10 mM KCl, 10 mM EDTA, 1 mM dithiothreitol, 0.4% [octylphenoxy] polyethoxyethanol, plus protease inhibitors) was added, and the plate was put on a rocking platform at 4 °C for 10 min. Cells were scraped from the plates, and cell clumps were disrupted by repetitive pipetting. The suspension was centrifuged at 15,000 x *g* at 4 °C for 5 min. The pellet was resuspended into 50 μl of buffer B (20 mM HEPES, pH 7.9, 200 mM NaCl, 1 mM EDTA, 10% glycerol, 1 mM dithiothreitol, plus protease inhibitors) by vigorous vortexing for 5 min and then at a medium vortex setting for 30 min at 4 °C. The suspension was centrifuged at 15,000 x *g* at 4 °C for 5 min, and the supernatant was collected (nuclear fraction). Equal amounts of protein extracts were subjected to immunoblot analysis and nuclear protein lamin B was used as internal control.

### Immunofluorescence and confocal microscopy

BMDM were fixed with paraformaldehyde (4%) at 37 °C for 20 min and then permeabilized with 0.2% Triton X-100 for 15 min. After blocking with 5% bovine serum albumin (BSA) with normal IgG (1:300) for 1 h, immunostaining was performed with primary antibodies against PARP-1 and TXNIP in 1% BSA overnight at 4 °C. After washing with PBS, cells were incubated with secondary antibodies in 1% BSA in PBS solution for 1 h at room temperature and then mounted with DAPI Fluoromount-G (SouthernBiotech, Birmingham, AL). Images were acquired using a Zeiss LSM780 confocal microscope (Carl Zeiss MicroImaging GmbH, 07440, Jena, Germany).

### Poly(ADP-ribosyl)ation in vitro

Purified recombinant rhPARP-1 and rhGST-NLRP3 were incubated in a mixture (25 μl) containing reaction buffer (100 mM Tris–HCl, 1 mM DTT, 10 mM MgCl_2_) with or without DPQ, sonicated salmon sperm DNA and 2 mM NAD (Sigma-Aldrich, St. Louis, MO, USA) for 30 min at 37 °C. The reaction was terminated by the addition of an equal volume of SDS sample buffer and heating at 95 °C for 5 min. Samples were then subjected to immunoblot analysis with antibody to PAR, NLRP3 or GST.

### LPS–mediated peritonitis

WT and *Parp-1*^−/−^ mice received intraperitoneal injection of PBS alone or 1 mg/kg of LPS. The influx of neutrophil into peritoneal cavity was determined 6 h later. The presence of IL-1β in serum was quantitated by ELISA.

### Statistical evaluation

The values were expressed as the mean ± SEM of at least three independent experiments that were performed in duplicate. A Student’s *t* test was used to determine whether the differences were statistically significant (*P* < 0.05).

## Results

### PARP-1 knockout decreases various stimuli-induced NLRP3 activation

**Fig. 1:**
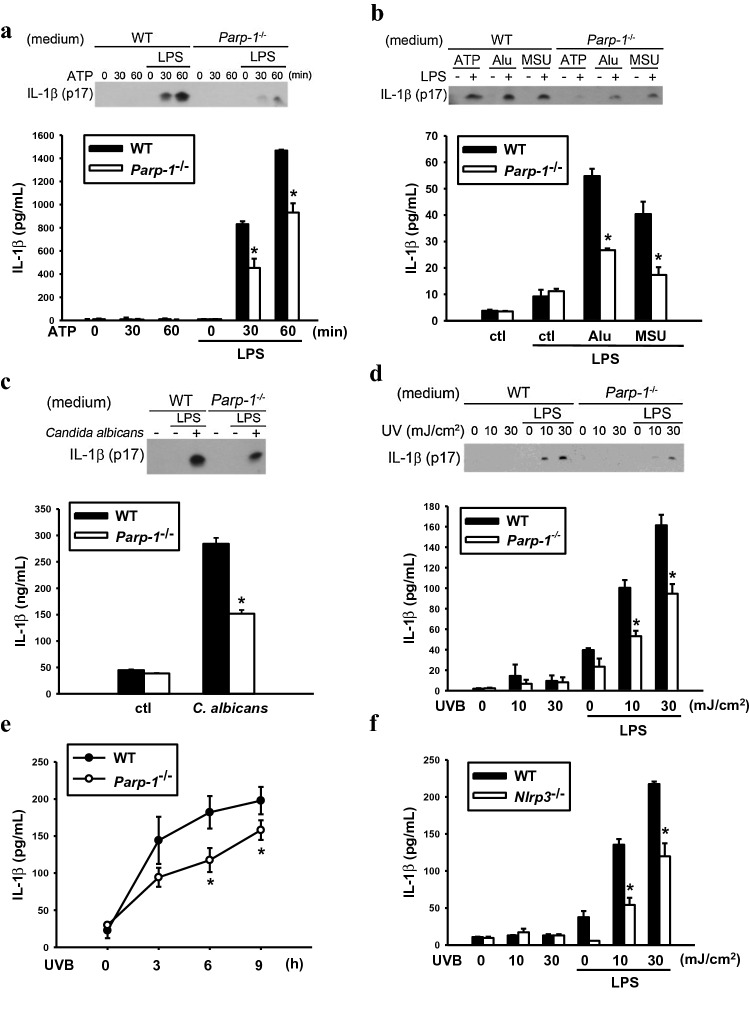
PARP-1 knockout decreases various stimuli-induced NLRP3 activation. Wild type (WT) and *Parp-1*^−/−^ BMDM were priming with LPS (1 μg/ml) for 6 h, then treated with ATP (5 mM) for the indicated time (**a**) or 30 min (**b**), Alu (150 μg/ml) or MSU (100 μg/ml) for 9 h (**b**), *Candida albicans* (MOI = 10) for 6 h (**c**), UVB with indicated dose for 6 h (**d**) or UVB (30 mJ/cm^2^) for indicated times (**e**). In **f** WT and *nlrp-3*^−/−^ BMDM were priming with LPS (1 μg/ml) for 6 h then irradiated with UVB (10, 30 mJ/cm^2^) with indicated dose and incubated for additional 6 h. The media were collected to determine the concentration of secreted IL-1β by western blot and ELISA. Data were means ± SEM from 3 independent experiments. *P < 0.05, when comparing IL-1β releasing from WT and *Parp-1*^−/−^ BMDM or WT and *Nlrp-3*^−/−^ BMDM

To understand the crucial role of PARP-1 in regulation of NLRP3 activation, we first used BMDM from WT  and *Parp-1*^*−/−*^ mice to investigate the involvement of PARP-1 in NLRP3 activators-induced IL-1β production. After LPS priming for 6 h, WT or *Parp-1*^*−/−*^ macrophages were treated with different NLRP3 activators, including ATP **(**Fig. 1a), aluminum gel (Alu) and MSU (Fig. 1b) for different times. Both ELISA assay and western blotting were performed for determining IL-1β release. Our results demonstrated that PARP-1 deficiency reduced the secretion of IL-1β caused by NLRP3 activators. *Candida albicans* has been reported as NLRP3 activator and can induce a robust production of IL-1β [30]. We also infected primary macrophages with *Candida albicans* and found that PARP-1 deletion can significantly block IL-1β release (Fig. 1c). UVB has been demonstrated to activate inflammasome in human keratinocytes [31]. Since macrophages also reside in dermis layer as an important immune cell type in skin, we also investigated whether UVB can be a NLRP3 activator in macrophages. In addition, because UVB exposure can activate PARP-1 as important DNA repair machinery, we would like to investigate whether PARP-1 can also participate in UVB-induced NLRP3 inflammasome activation. In LPS-primed BMDM, we found that UVB can induce IL-1β secretion in dose-dependent and time-dependent manners and this effect was decreased in *Parp-1*^−/−^ BMDM (Fig. 1d and e) and also in *Nlrp3*^*−/−*^ BMDM (Fig. 1f). These findings suggest that UVB-induced IL-1β secretion in BMDM is PARP-1- and NLRP3-dependent. To understand if such inhibition of IL-1β secretion in *Parp-1*^*−/−*^ macrophages might be associated with cell viability, we performed LDH assay. We found that there is no difference of cell viability between WT and *Parp-1*^−/−^ BMDM upon stimulation with these NLRP3 activators, ruling out the decreased IL-1β release is due to cell death (Fig. S1a).

### PARP deficiency attenuates caspase-1 activation and decreases NLRP3 inflammasome assembly with no effect on NLRP3 complex expression

**Fig. 2:**
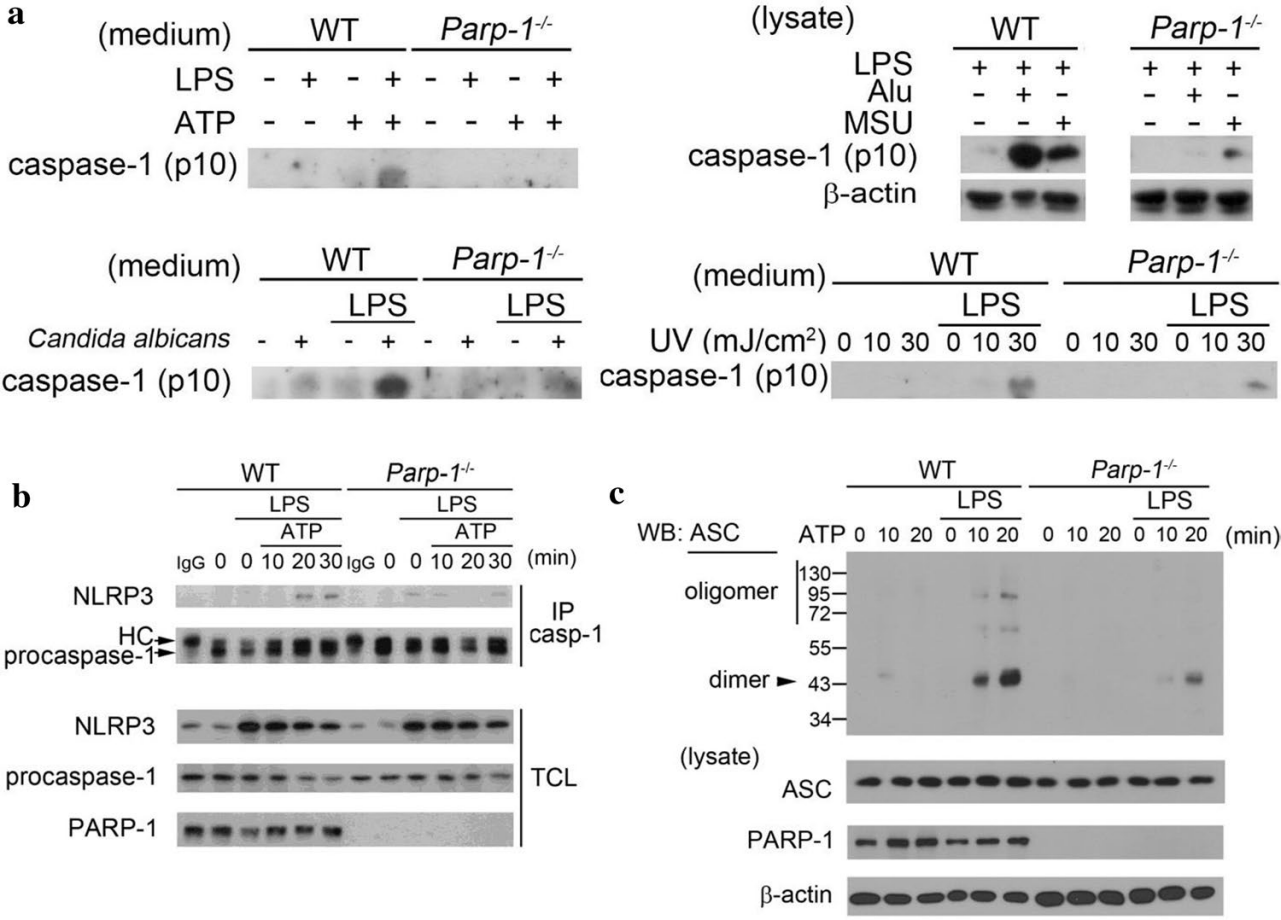
PARP deficiency attenuates caspase-1 activation and decreases NLRP3 inflammasome assembly. **a** WT and *Parp-1*^−/−^ BMDM were primed with LPS (1 μg/ml) for 6 h, and then treated with ATP (5 mM) for 30 min (uppper left), Alu (150 μg/ml) or MSU (100 μg/ml) for 6 h (upper right), infected with *Candida albicans* (MOI = 10) for 3 h (lower left) or irradiated with indicated dose of UVB for 6 h (lower right). Media or total cell lysates were analyzed for cleaved caspase-1 p10 and β-actin by immunoblotting. **b** WT and *Parp-1*^−/−^ BMDM were primed with LPS (1 μg/ml) for 6 h then stimulated with ATP (5 mM) for indicated time. Total cell lysates were collected for immunoprecipitation by caspase-1 antibody. Samples were analyzed by immunoblotting for antibodies specific against NLRP3, caspase-1 and PARP-1. **c** After priming for 6 h, WT and *Parp-1*^−/−^ BMDM were stimulated with ATP (5 mM) for indicated time. Total cell lysates were collected for ASC oligomerization assay and samples were analyzed by immunoblotting for detecting the oligomer and dimer of ASC

Since NLRP3 inflammasome fully activation requires two steps, we investigated which step PARP-1 is involved in. We found LPS-induced pro-IL-1β expression at both mRNA (Fig. S2a left panel) and protein (Fig. S2b) levels were the same between WT and *Parp-1*^*−/−*^ BMDM. In addition, PARP-1 deficiency did not affect the mRNA and protein induction of NLRP3, ASC or pro-caspase-1 (Fig. S2a right panel and Fig. S2b), indicating that PARP-1 does not regulate IL-1β release by decreasing the step 1. In contrast, the processing of pro-caspase-1 to mature caspase-1 (p10) and release to medium caused by ATP, Alu and MSU were decreased in *Parp-1*^*−/−*^ macrophages (Fig. 2a). *Candida albicans* infection- and UVB irradiation-induced caspase-1 maturation were also decreased in *Parp-1*^*−/−*^ BMDM (Fig. 2a). The decreased processing of caspase-1 suggests that PARP-1 may get involved in the step 2 activation of NLRP3 inflammasome.

Since PARP-1 gets involved in NLRP3 inflammasome activation by regulating caspase-1 maturation, we next determined whether PARP-1 can regulate the inflammasome complex formation. We found that the interaction of NLRP3 with pro-caspase-1 was decreased in *Parp-1*^*−/−*^ BMDM (Fig. 2b), indicating that PARP-1 can regulate inflammasome complex assembly. In addition, ASC cross-linking assay also showed that PARP-1 deficiency can decrease ASC oligomerization (Fig. 2c). Overall, these data indicate that PARP-1 participates in the step 2 for caspase-1 maturation.

### PARP activator and inhibitor oppositely regulate NLRP3 activation

**Fig. 3:**
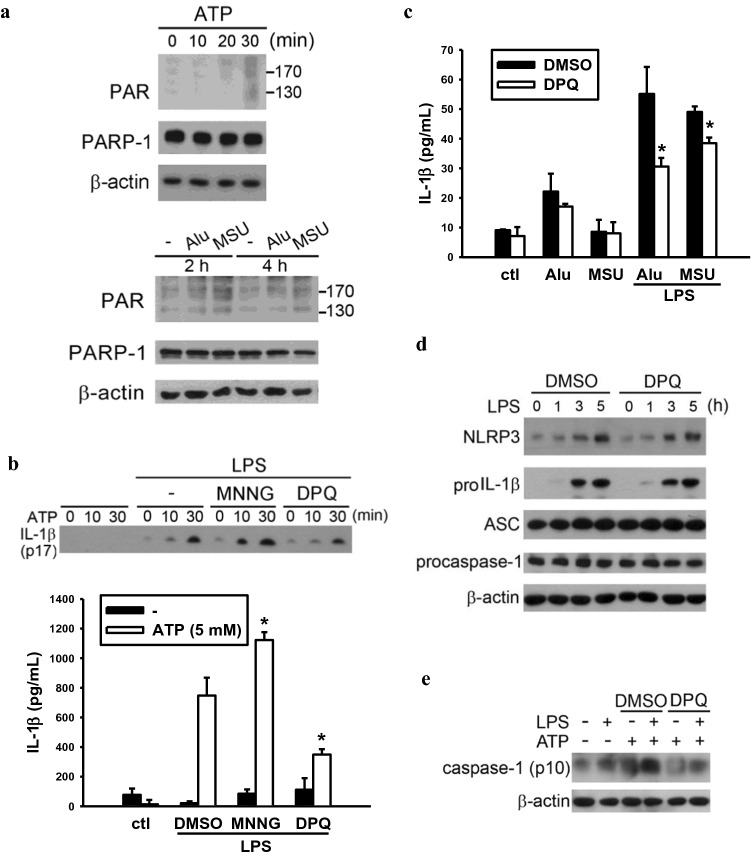
PARP activator and inhibitor oppositely regulate NLRP3 activation. **a** WT BMDM were treated with ATP (5 mM) (upper panel) or Alu (150 μg/ml), MSU (100 μg/ml) (lower panels) for indicated time. Total cell lysates were subjected to immunoblotting for PAR, PARP-1 and β-actin antibodies. **b** WT BMDM were primed with LPS (1 μg/ml) for 6 h, then pretreat with MNNG (100 μM) or DPQ (25 μM) for 30 min. ATP (5 mM) stimulated BMDM subsequently for 30 min then collect media to determine the concentration of secreted IL-1β by western blot and ELISA. **c** WT macrophages were primed with LPS (1 μg/ml) for 6 h, then pretreated with DPQ (25 μM) for 30 min. Alu (150 μg/ml) or MSU (100 μg/ml) stimulated cell for 9 h subsequently then collect media for analyzing IL-1β release by ELISA. **d** WT macrophages were pretreated with DPQ (25 μM) for 30 min then stimulated with LPS (1 μg/ml) for indicated time. Total cell lysates were analyzed by western blot for the expression of inflammasome-associated proteins. **e** WT macrophages were primed with LPS (1 μg/ml) for 6 h then pretreated with DPQ (25 μM), followed by ATP (5 mM) stimulation for 30 min. Total cell lysates were subjected to immunoblotting for analyzing the expression of caspase-1 p10 and normalized by β-actin expression. Data were means ± SEM from 3 independent experiments. *P < 0.05, when comparing IL-1β releasing from DMSO and MNNG or DPQ-treated group

Given that PARP-1 participates in NLRP3 activator-induced IL-1β release, we would like to further dissect whether PARP-1’s enzyme activity is required for regulating NLRP3 activation. We observed that ATP, Alu and MSU treatment can stimulate PAR formation, indicating the increase of PARP-1 activity (Fig. 3a). MNNG is a well-known alkylating agent which can cause DNA strands break then leading to strong PARP-1 activation [32]. We found that MNNG treatment after LPS priming can enhance ATP-induced IL-1β release. On the other hand, PARP-1 inhibitor, DPQ, can decrease ATP-induced IL-1β release (Fig. 3b). Other NLRP3 activators Alu- and MSU-induced IL-β release can also be decreased by DPQ (Fig. 3c). However, PARP-1 enzymatic inhibition did not alter the protein expressions of NLRP3, ASC, pro-caspase-1 and proIL-1β (Fig. 3d). After LPS priming, pretreatment of DPQ also attenuated the cleavage of pro-caspase-1 to active form p10 (Fig. 3e). All these results confirm our data in *Parp-1*^*−/−*^ BMDM and suggest that PARP-1 activation is involved in the step 2 for NLRP3 inflammasome activation.

### PARP-1 deficiency decreases ROS production

**Fig. 4:**
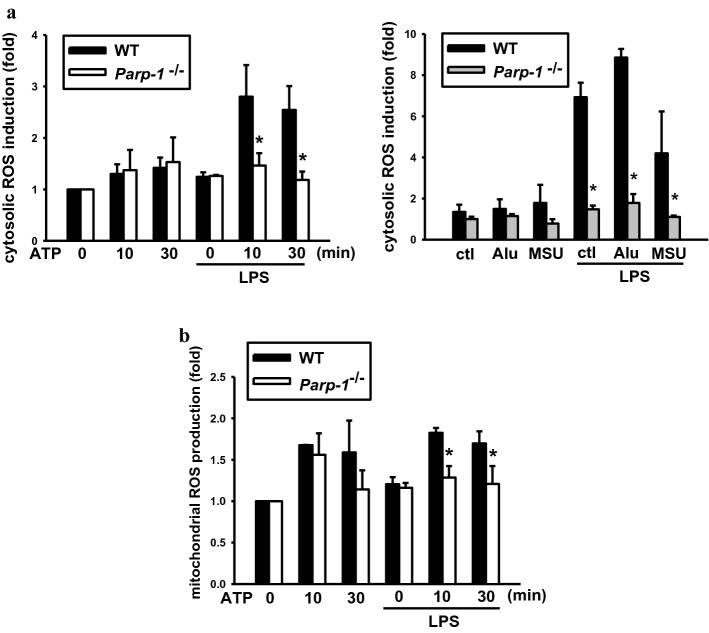
PARP-1 deficiency decreases ROS production. **a** WT and *Parp-1*^−/−^ BMDM were primed with LPS (1 μg/ml) for 6 h then stimulated with ATP (5 mM) (left panel) for indicated time or Alu (150 μg/ml) or MSU (100 μg/ml) for 9 h subsequently (right panel). ROS induction was detected by CM-H2DCFDA and normalized by each untreated control group. **b** After priming by LPS (1 μg/ml) for 6 h, WT and *Parp-1*^−/−^ BMDM were treated with ATP (5 mM) for indicated time. Mitochondrial ROS production was stained by MitoSox and normalized by each untreated control group. Data were means ± SEM from 3 independent experiments. *P < 0.05, when comparing ROS production between WT and *Parp-1*^−/−^ BMDM

ROS can be generated by all known NLRP3 activators to activate inflammasome [18, 19]. Upon oxidative stress-induced DNA damage, PARP-1 can be activated by ROS and reciprocally further increase ROS production [33]. As we observed the decreased inflammasome complex formation in *Parp-1*^*−/−*^ BMDM, we wondered whether PARP-1 also plays a role in NLRP3 activators-induced ROS production. Our data showed a decreased cellular  ROS production caused by ATP, Alu and MSU in LPS-primed *Parp-1*^−/−^ cells (Fig. 4a). We also found that ATP-induced mitochondrial ROS production was also attenuated in LPS-primed *Parp-1*^*−/−*^ BMDM (Fig. 4b). Overall, these data correlate to our observation that PARP-1 can regulate NLRP3 activation at signal 2.

### ROS induce cytosolic translocation of PARP-1 and the interaction of PARP-1 with TXNIP and NLRP3

**Fig. 5:**
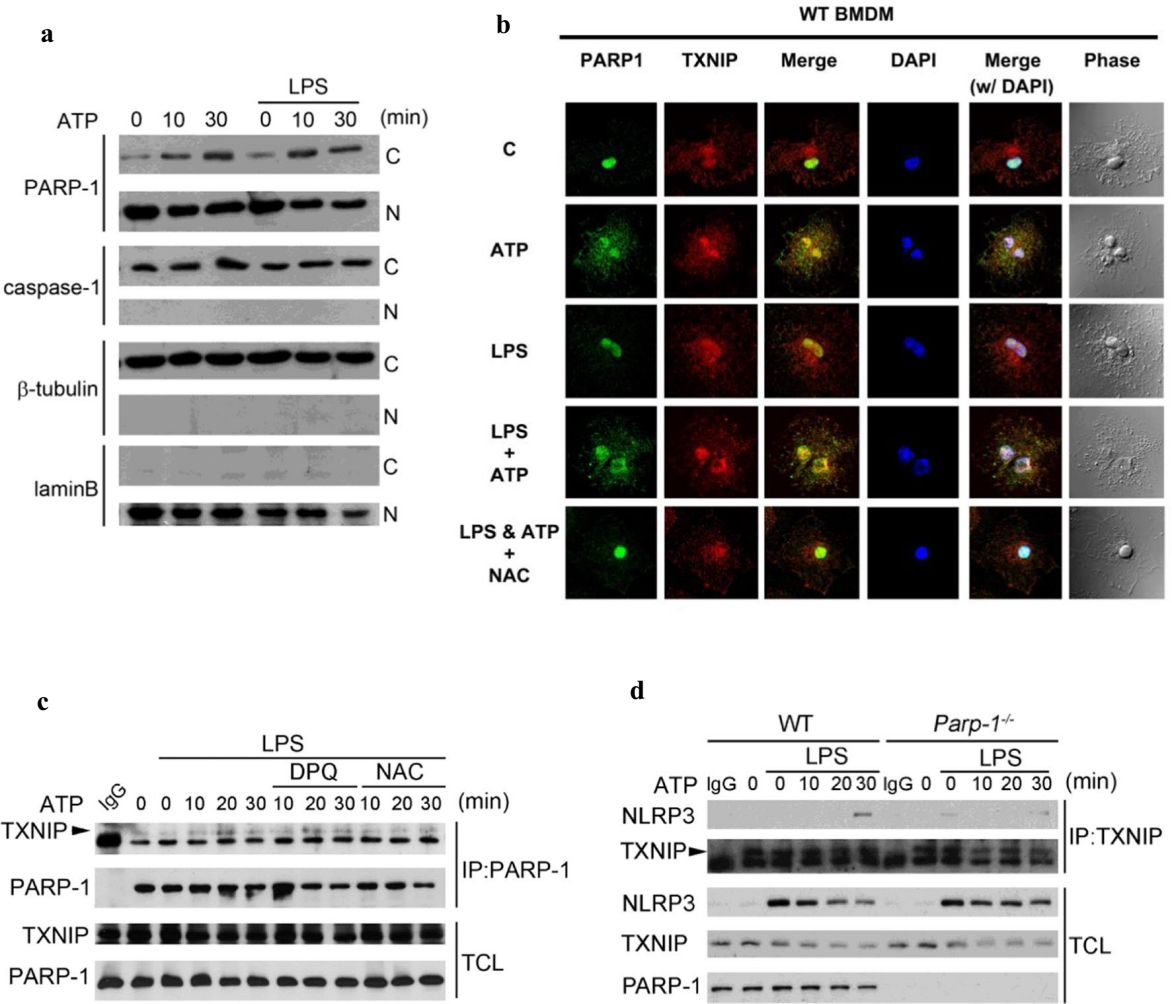
ROS induce cytosolic translocation of PARP-1 and the interaction of PARP-1 with TXNIP and NLRP3. **a** WT macrophages were primed with LPS (1 μg/ml) for 6 h. ATP (5 mM) stimulated for the indicated time subsequently. Total cell lysates were collected for nuclear fractionation and subjected to western blot for detecting of PARP-1 and pro-caspase-1. β-Tubulin and lamin B were the markers also internal control of cytosolic and nuclear fractions, respectively. **b** WT macrophage were primed with LPS (1 μg/ml) for 6 h and treated with ATP (5 mM) for 10 min then fixed and subjected to immunostaining with PARP-1 and TXNIP antibodies. Images were acquired using a Zeiss LSM780 confocal microscope. **c** WT macrophages were first primed with LPS (1 μg/ml) for 6 h, following by pretreatment of DPQ (25 μM) or NAC (1 mM) for 30 min then stimulated with ATP (5 mM) for indicated time. Total cell lysates were collected for immunoprecipitation by PARP-1 antibody. Samples were analyzed by immunoblotting for antibodies specific against TXNIP and PARP-1. **d** WT and *Parp-1*^−/−^ BMDM were primed with LPS (1 μg/ml) for 6 h then stimulated with ATP (5 mM) for indicated time. Total cell lysates were collected for immunoprecipitation by TXNIP antibody. Samples were analyzed by immunoblotting for antibodies specific against NLRP3, TXNIP and PARP-1

PARP-1 has been reported as a nuclear protein to execute the function of DNA repair. Because our above results indicate the involvement of PARP-1 in NLRP3 inflammasome activation which mainly resides in cytosol, we would like to investigate whether NLRP3 activators can stimulate PARP-1 translocation from nuclei to cytosol. We fractionated the cell lysate of BMDM and found that cytosolic PARP-1 level was increased under ATP treatment (Fig. 5a). In contrast, caspase-1 remained localization in cytosol fraction. Immunostaining data also revealed that ATP stimulation alone can induce PARP-1 translocation to cytosol, while LPS itself has no such effect (Fig. 5b). We also showed that TXNIP was present in the nuclei and cytosol at resting state by immunostaining. Interestingly, nuclear TXNIP and PARP-1 were moderately co-localized and LPS stimulation can markedly increase their association in the nuclei (Fig. 5b). Moreover, we observed the interaction between PARP-1 and TXNIP in the cytosol and nuclei upon ATP stimulation. These effects of ATP were still observed in LPS-priming cells but were decreased by ROS scavenger NAC (Fig. 5b). These results suggest that ATP-induced ROS production leads to the cytosolic translocation of PARP-1 and its association with TXNIP in cytosol.

After observing PARP-1 can translocate to cytosol and regulate NRLP3 inflammasome complex assembly, we were curious whether PARP-1 can be recruited in inflammasome complex. After immunoprecipitation of PARP-1, we found the association of PARP-1 and NLRP3 after ATP stimulation, and the interaction was decreased by NAC (Fig. 5c). Of note, PARP-1 inhibitor did not affect the association between TXNIP and PARP-1 (Fig. 5c). Moreover, confirming previous finding that TXNIP association with NLRP3 contributed to NLRP3 assembly [18], our immunoprecipitation data revealed the increased TXNIP-NLRP3 association after ATP stimulation in WT cells. Interestingly, this interaction was decreased in *Parp-1*^*−/−*^ BMDM (Fig. 5d). From these results we suggest that PARP-1 may get involved in NLRP3 and TXNIP association, subsequently regulating NLRP3 inflammasome complex formation.

### PARP-1 can interact with TXNIP and NLRP3 and mediate NLRP3 PARylation

**Fig. 6:**
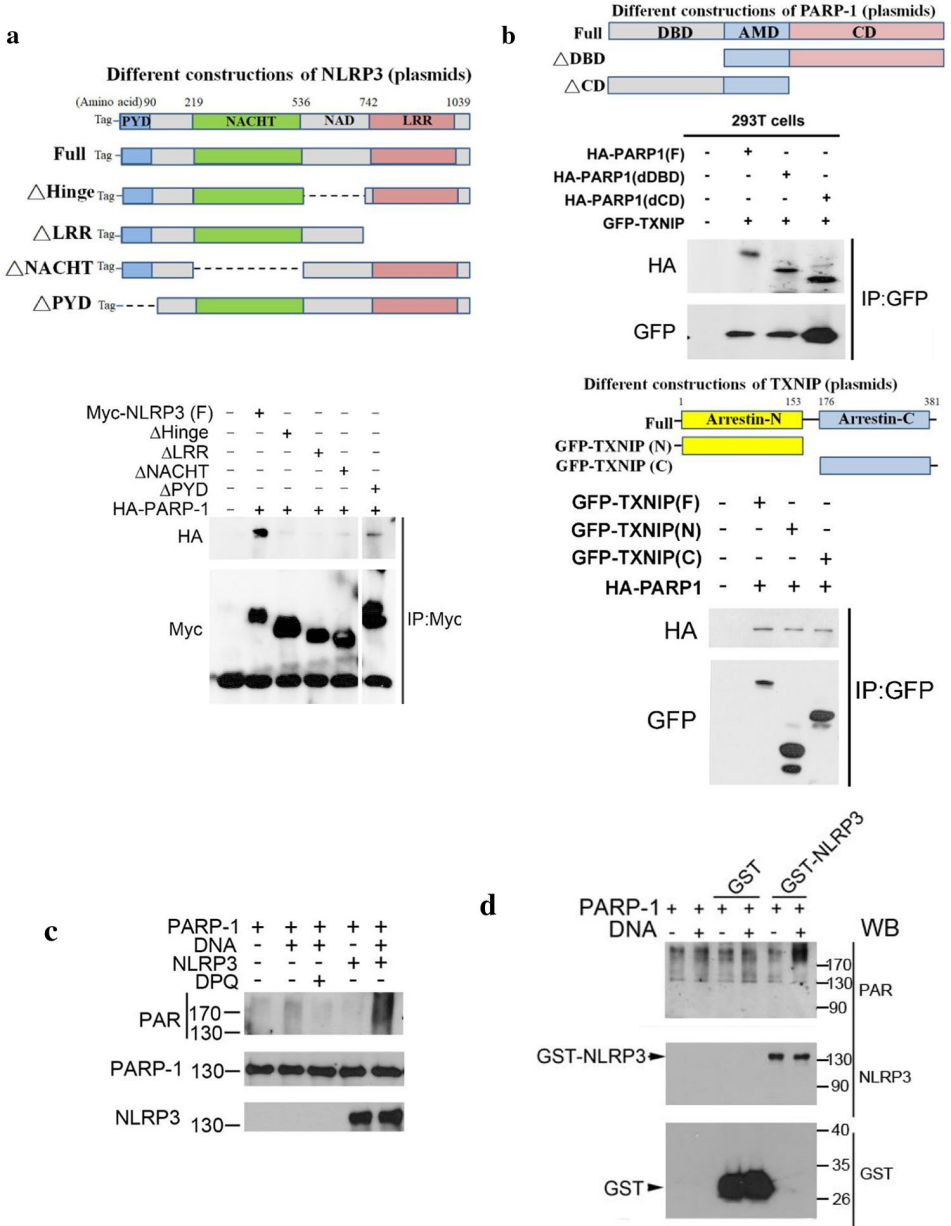
PARP-1 binds to TXNIP and NLRP3 and mediates PARylation of NLRP3. **a** HEK293T cells were co-transfected with plasmid encoding HA-tagged PARP-1 together with plasmids encoding the indicated Myc-tagged NLRP3-deletions. Twenty-four hours later, cell lysates were subjected to immunoprecipitation with an anti-Myc antibody and were analyzed by Western blotting with antibodies against the HA or Myc as indicated. The schematic diagram of Myc-tagged NLRP3 constructs includes full length (F), Hinge domain deleted (dHinge), LRR domain deleted (dLRR), NATCH domain deleted (dNATCH) and PYD domain deleted (dPYD). **b** HEK293T cells were co-transfected with plasmids encoding GFP-tagged TXNIP together with plasmids encoding the indicated HA-tagged PARP-1 deletions (upper panel) or plasmids encoding HA-tagged PARP-1 together with GFP-tagged TXNIP deletions (lower panel). Twenty-four hours later, cell lysates were subjected to immunoprecipitation with an anti-GFP antibody and were analyzed by Western blotting with antibodies against the HA and GFP as indicated. The schematic diagram of HA-tagged PARP-1 constructs includes full length (F), DNA binding domain deleted (dDBD) and catalytic domain deleted (dCD). The schematic diagram of GFP-tagged TXNIP constructs includes full length (F), C-terminus deleted (N) and N-terminus deleted (C). In **c** and **d**, recombinant proteins including GST, PARP-1 and GST tagged-NLRP3 were performed PARylation assay. PARP-1 inhibitor DPQ (25 μM) was added as negative control of the assay. Samples were analyzed by immunoblotting of antibodies specific against PAR, PARP-1, GST and NLRP3

To further investigate the interaction between PARP-1, NLRP3 and TXNIP, we conducted overexpression experiment in HEK293T cells and conducted immunoprecipitation to dissect the interaction domains between NLRP3 and PARP-1. First, we indeed found the direct interaction between PARP-1 and NLRP3 upon overexpressing full construct of each protein. In constructs with deletions of various domains of NLRP3, only PYD domain deletion form of NLRP3 can still interact with PARP-1, indicating that the NATCH, NAD and LRR domains of NLRP3 all are required for the interaction with PARP-1 (Fig. 6a). Similar deletion approach was applied to PARP-1 and TXNIP, and we found PARP-1 can interact with TXNIP. Deletion of PARP-1 at either N-terminal DNA binding domain (DBD) or C-terminal catalytic domain (CD) still can bind TXNIP with similar extent. On the other hands, TXNIP containing only arrestin-N or arrestin-C domain was sufficient to bind PARP-1 (Fig. 6b). These findings suggest that the interaction between PARP-1 and TXNIP requires automodification domain (AMD) of PARP-1 and arrestin domain of TXNIP.

PARylation is an important post-translational modification, and can regulate protein–protein interaction [25, 34, 35]. Since PARP-1 got involved in NLRP3 inflammasome assembly, we would like to investigate whether the components of inflammasome might be target(s) of PARP-1. We performed in vitro PARylation assay as previously described [36] by incubation of PARP-1 and GST-NLRP3 recombinant proteins as well as sheared salmon DNA to activate PARP-1. In this assay, DNA presence can induce PARP-1 auto-PARylation and this effect was blocked by DPQ. Of note, the addition of GST-NLRP3 can enhance the signal intensity of PAR (Fig. 6c), suggesting that PARP-1 may also modify NLRP3. Because we used GST fusion protein of NLRP3 in this enzymatic reaction, we needed to check if GST rather than NLRP3 might be the target of PARP-1. To clarify this concern, we determined the PAR signal of GST protein in this assay. As shown in Fig. 6d, GST-NLRP3 can be PARylated, while GST cannot. Overall, we proposed that PARP-1 can PARylate NLRP3 then promote the protein interaction of inflammasome complex.

### PARP-1 deficiency decreases LPS-triggered peritonitis

**Fig. 7:**
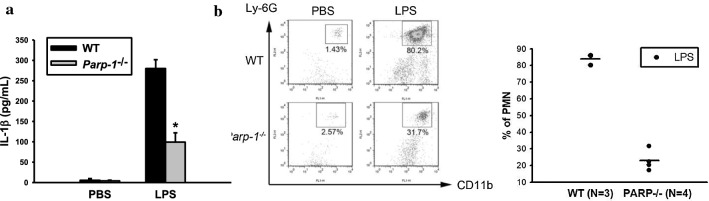
PARP-1 deficiency decreases LPS-triggered peritonitis. **a** PBS (0.5 ml) or LPS (1 mg/kg) was intraperitoneally administered to control and *Parp-1*^−/−^ mice. Mice were sacrificed 6 h later, and levels of IL-1β in serum were determined by ELISA. **b** The population of neutrophils in peritoneal lavage was assessed by staining with anti-Ly-6G and anti-CD11b. Numbers indicate percentages of polymorphonuclear neutrophils (PMN). *P < 0.05, when comparing serum IL-1β level between WT and *Parp-1*^−/−^ mice

To verify above findings of PARP-1 in mediating NLRP3 activation in animals, we used the peritonitis model in WT and *Parp-1*^−/−^ mice. This is because IL-1β is a potent proinflammatory cytokine to cause neutrophil recruitment to the inflammatory loci, and plays a crucial role in the initiation step of gout triggered by MSU [22, 37]. Mice were received intraperitoneal injection of PBS or LPS. The influx of neutrophils into the peritoneal cavity and the presence of IL-1β were determined subsequently. As shown in Fig. 7a, a lower quantity of IL-1β production was detected in *Parp-1*^−/−^ mice receiving LPS compared with those measured in the control group. In addition, peritoneal infiltration of polymorphonuclear leukocytes (PMN) was reduced by 50% in *Parp-1*^−/−^ mice (Fig. 7b). These results support a critical function of PARP-1 in inflammasome activation in vivo.

## Discussion

Besides from DNA repair function, PARP-1 also plays versatile roles in mediating innate immunity and adaptive immune responses [38–40]. NLRP3 inflammasome is a pivotal sensor of innate immune response. Our work demonstrates a novel mechanism to regulate NLRP3 inflammasome by PARP-1. In this study, we for the first time demonstrate that except phosphorylation and ubiquitination, PARylation is a new way to regulate NLRP3 inflammation activation, and plays a crucial step in protein–protein interaction. We found that nuclear PARP-1 can translocate to cytosol and regulate NLRP3 inflammasome activation by directly targeting NLRP3. This event in turn mediated the interaction of NLRP3 and TXNIP thus affecting inflammasome complex formation.

Previous studies have shown two kinds of post-translational modification that can regulate inflammasome activity. One is the phosphorylation of NLRP3 [41], NLRC4 [42] and ASC [29] and they are required for inflammasome activation [42, 43]. The other is the deubiquitination of NLRP3 by deubiquitinase BRCC3 and it is critical for NLRP3 activation [44]. NLRP3 has been shown to be ubiquitinated in resting macrophages and these ubiquitin chains are removed by deubiquitinase upon cell activation with LPS priming following activating signals (ATP, nigericin and MSU crystals), allowing the activation of the inflammasome complex. Here our work further provides a unique and different type of modification of inflammasome component that NLRP3 poly-ADP-ribosylation by PARP-1 contributes to inflammasome activation. First, we found that NLRP3 activators can also induce PAR complex formation (Fig. 3a), implying the up-regulation of PARP-1 activity upon NLRP3 activation. Second, enzymatic inhibition of PARP-1 by DPQ can decrease NLRP3 activation-induced IL-1β release (Fig. 3b, c), indicating that PARP-1 activity positively regulates NLRP3 activation. In vitro PARylation assay also showed that NLRP3 can be PARylated by PARP-1 (Fig. 6c, d). Interestingly, previous studies done by Bose et al. showed that NLRP3 can be ADP-ribosylated by *Mycoplasma pneumoniae* CARDS toxin and the ADP-ribosyltransferase activity of CARDS toxin is essential for NLRP3 inflammasome activation [45]. Due to the negative charged structure, ADP-ribosylation has been shown to regulate protein–protein interaction [25, 34]. Therefore, it is interesting to further determine other PARylation-associated molecules and regulating events for NLRP3 inflammasome assembly and activation in the future. Currently we have not determined the potential targeted amino acids of NLRP3 for PARylation, but it is an interesting issue for future work. We are also interested to understand if possible TXNIP is an additional target protein of PARP-1.

In this study, we also demonstrate PARP-1-dependent NLRP3-TXNIP interaction which is regulated by ROS. Oxidative stress has been considered as a critical signal in the activation of inflammasome and TXNIP is an important link since the binding of TXNIP with NLRP3 was shown to promote inflammasome activation [18]. By performing immunoprecipitation with PARP-1 antibody, we found that PARP-1 can interact with NLRP3 and also TXNIP upon ATP stimulation (Fig. 5c, d). PARP-1 may regulate the association of NLRP3 and TXNIP in inflammasome complex because PARP-1 deficiency can decrease their interaction (Fig. 5d). In previous study, PARP-1 has been demonstrated to interact with TXNIP in human umbilical vein endothelial cells in basal condition [46]. It seems that PARP-1 activity can modulate the localization of TXNIP. They found PARP-1 inhibitor, PJ34, can decrease autoPARylation of PARP-1 and inhibit PARP-1’s association with TXNIP. PARP-1 inhibition enables the translocation of TXNIP from nucleus to plasma membrane, which subsequently leads to stimulate VEGFR2 signal and promotes endothelial cells survival upon apoptotic stress. From this work we strengthen the notion that post-translation modification of proteins by PARylation serves as an important mechanism in regulating cellular signaling and functions. In this aspect, previous study also showed that PARP-1-mediated PARylation of p65 promotes NK-κB-dependent inflammatory gene expression in macrophage [47]. Moreover, PARrylation of p53 or p65 by PARP-1 hinders their interaction with nuclear export receptor Crm1 (chromosomal region maintenance 1) thus enhancing their nuclear retention [48, 49]. Therefore, PARylation becomes an important mechanism to regulate subcellular localization and functions of proteins.

On the other hand, PARP-1 may also have impact on the oxidative status upon NLRP3 inflammasome activation hence regulating NLRP3 and TXNIP interaction. We found lower ROS induction in PARP-1 knockout BMDM after NLRP3 agonist stimulation (Fig. 4a, b). Previous study demonstrated that ROS induction is required for the dissociation of TXNIP from TRX and promotes the binding of TXNIP with NLRP3 subsequently [18]. In line with our finding, we also observed a decreased interaction of TXNIP and NLRP3 in *Parp-1*^−/−^ BMDM compared to WT group (Fig. 5d). Intriguingly, our previous work also showed that PARP-1 can positively regulate UVB-induced ROS production in keratinocytes which is partially resulting from EGFR transactivation [33]. Another study mentioned that PARP-1 activation by oxidative stress contributes to the impairment of mitochondrial integrity by decreasing MKP-1 expression in an ATF4-dependent manner then activating p38 and JNK, leading to mitochondrial ROS production [50]. In this study, we also found that ATP treatment can induce mitochondrial fission, but this effect was not changed by PARP-1 KO (data not shown), excluding the effect of PARP-1 in regulation of mitochondrial ROS production upon ATP treatment in LPS-primed macrophages is related to mitochondrial morphology.

Although PARP-1 is a nuclear protein, it has been shown to be translocated to cytoplasm [51–53] or localized to mitochondria [54]. Here we also observed that PARP-1 can translocate to cytoplasm upon ATP stimulation (Fig. 5a, b). Notably, the translocation of PARP-1 can be blocked by ROS scavenger (Fig. 5b), suggesting that ROS induction is a required signal in PARP-1 translocation. Since ROS induction is important in step 2 signal for NLRP3 inflammasome activation [55], our data imply that PAPR-1 translocation maybe a downstream event of ROS induction. Previous study has mentioned that leptomycin-B, a Crm1 inhibitor, can inhibit NLRP3 activation-induced IL-1β release [56]. These data suggest that nuclear protein exportation may participate in inflammasome activation, while the molecular mechanism remains unknown. Our data suggest that PARP-1 exported to cytoplasm by NLRP3 activating stimuli can interact with other inflammasome complex proteins like NLRP3 and TXNIP therefore promoting inflammasome activation. However, microorganism may also manipulate the PARP-1 localization to control host cell immune response. Muthumani et al. discovered that HIV protein Vpr can use glucocorticoid receptor as the recruitment vehicle to transport PARP-1 to cytosol, preventing nuclear PARP-1 to activate NF-κB signal for host defense [57].

As in apoptosis cleaved by caspase-3, PARP-1 has been shown to be cleaved by caspase-7 and caspase-1 upon NLRP3- or NLRC4-induced pyroptosis [58]. Because PARP-1 activation can consume ATP, the precursor of PARP-1’s substrate NAD^+^, proteolytic inactivation of PARP-1 is considered as an energy restriction strategy during the execution of cell death. Moreover, Erener’s work also indicates that LPS (10 μg/ml)-induced caspase-7 activation can cleave PARP-1 in peritoneal macrophages and this effect is NLRP3- and caspase-1-dependent [59]. The cleaved PARP-1 leads to dissociation of PARP-1 from promoters of a subset of NF-κB target genes that are negatively regulated by PARP-1, thus turning on the gene transcription. However, we do not observe significant cleavage of PARP-1 upon LPS (1 μg/ml) priming and NLRP3 stimulation. Indeed, no effect of LPS on PARP-1 protein expression was also reported before in macrophages [60]. We speculate the findings of Erener et al. might be due to the much higher concentration of LPS used, allowing LPS going into cells to induce noncanonical NLRP3 activation and caspases-dependent pyroptosis and PARP-1 cleavage [61]. We found both genetic knockout and pharmacologic inhibition of PARP-1 are able to decrease NLRP3 activation-induced IL-1β release (Figs. 1 and 3b, c), indicating that PARP-1 is a novel mechanism to positively regulate NLRP3 and caspase-1 activation other than the passive role of cleavage by caspase-1 or caspase-7. Observing the decreased caspase-1 maturation (Figs. 2a, 3e) and ASC oligomerization (Fig. 2c), but unaltered step 1 signal induction (Fig. S2 and 3d), we suggest the involvement of PARP-1 activity in NLRP3 inflammasome complex activation.

**Fig. 8:**
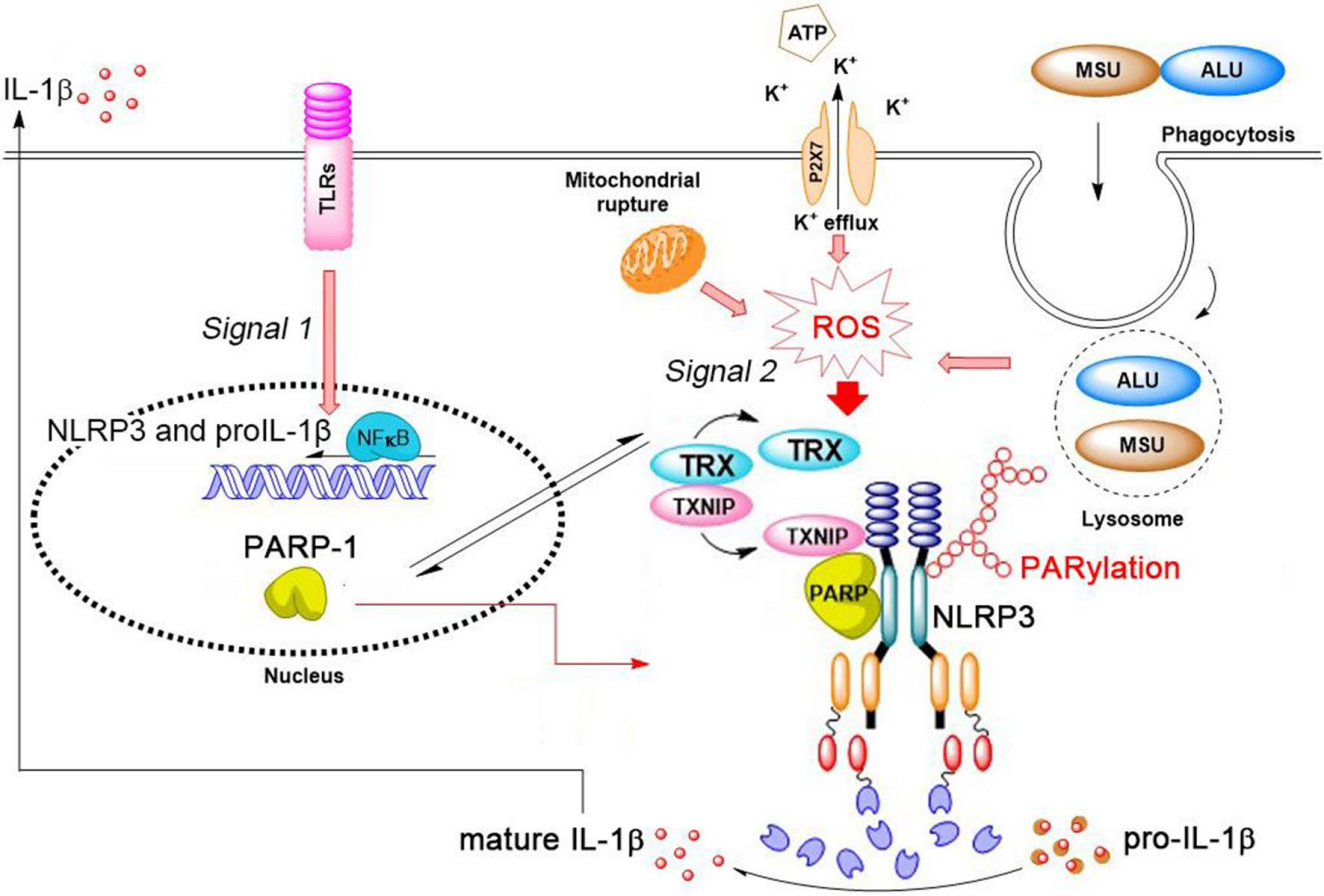
Schematic diagram of PARP-1 in regulation of NLRP3 inflammasome activation. Multiple NLRP3 stimulators like danger signals, environmental stimuli and pathogens activate the second step of NLRP3 activation may through ROS induction. Nuclear PARP-1 can export from nuclei to cytosol upon ROS production and catalyze the post-translation modification on NLRP3 by PARylation. Moreover, PARP-1 serves as the bridge of NLRP3 and TXNIP interaction for inflammasome activation. PARP-1 also involves to positively regulate ROS induction by NLRP3 stimuli. Overall, PARP-1 positively regulates NLRP3 inflammasome activation via NLRP3 PARylation, interaction between TXNIP and NLRP3, and ROS production

In conclusion as shown in the summary Fig. 8, we propose a novel mechanism of PARP-1 in regulation of NLRP3 inflammasome activation. Upon P2X7 activation by ATP, ROS production leads to nuclear PARP-1 activation and translocation from the nuclei to cytosol, where PARP-1 is recruited to NLRP3 inflammasome to induce post-translational PARylation of NLRP3. Moreover, the ROS induction also dissociates TXNIP from TRX and promotes TXNIP binding to NLRP3 via PARP-1. Both PARylation of NLRP3 and assembly with TXNIP increase NLRP3 inflammasome activation. In addition, PARP-1 and ROS reciprocally exert a positive regulation upon oxidative stress, which contributes to NLRP3 inflammasome activation. Facing numerous diseases mediated by excessive inflammasome activation and overproduction of IL-1β, the findings of our study provide evidence that PARP-1 is a novel target for drug development to reduce inflammasome-related diseases.

## Supplementary Information

Below is the link to the electronic supplementary material.

Supplementary file1 (DOCX 1293 KB)

## Data Availability

All data generated or analysed during this study are included in this published article and supplementary information files.

## Abbreviations

ASC: Apoptosis-associated speck-like protein containing a CARD domain
BMDM: Bone marrow-derived macrophages
ELISA: Enzyme-linked immunosorbent assay
IL-1β: Interleukin-1β
LDH assay: Lactate dehydrogenase assay
LPS: Lipopolysaccharide
MNNG: N-methyl-N'-nitro-N'-nitrosoguanidine
MTT: 3-(4,5-Dimethylthiazol-2-yl)-2,5-diphenyltetrazolium bromide
MSU: Monosodium urate
NAC: *N*-Acetyl-L-cysteine
PAR: Poly(ADP-ribose)
PARP-1: Poly(ADP-ribose) polymerase-1
PBS: Phosphate buffered saline
PMN: Polymorphonuclear leukocytes
ROS: Reactive oxygen species
TRX: Thioredoxin
TXNIP: Thioredoxin-interacting protein
UVB: Ultraviolet B
