# Supplementary material for: PARP-1 regulates inflammasome activity by poly-ADP-ribosylation of NLRP3 and interaction with TXNIP in primary macrophages
Source: Cell Mol Life Sci. 2022 Jan 30;79(2):108. doi: 10.1007/s00018-022-04138-z (PMC8801414; doi:10.1007/s00018-022-04138-z)
Supplement: Supplementary file 1 — Supplementary file1 (DOCX 1293 KB) [file 18_2022_4138_MOESM1_ESM.docx]

**Supplementary Information**

**Figure S1**

**b**

**a**

**Figure S1**

Wild type (WT) and *Parp-1*^-/-^ BMDM were priming with LPS (1 μg/ml) for 6 h, then treated with ATP (5 mM) for 30 min (a), Alu (150 μg/ml) or MSU (100 μg/ml) for 9 h (b) then medium were assayed by LDH assay. Cell death percentage was normalized by total cell lysis O.D. value. Data were means ± SEM from 3 independent experiments.

**Figure S2**

**b**

**a**


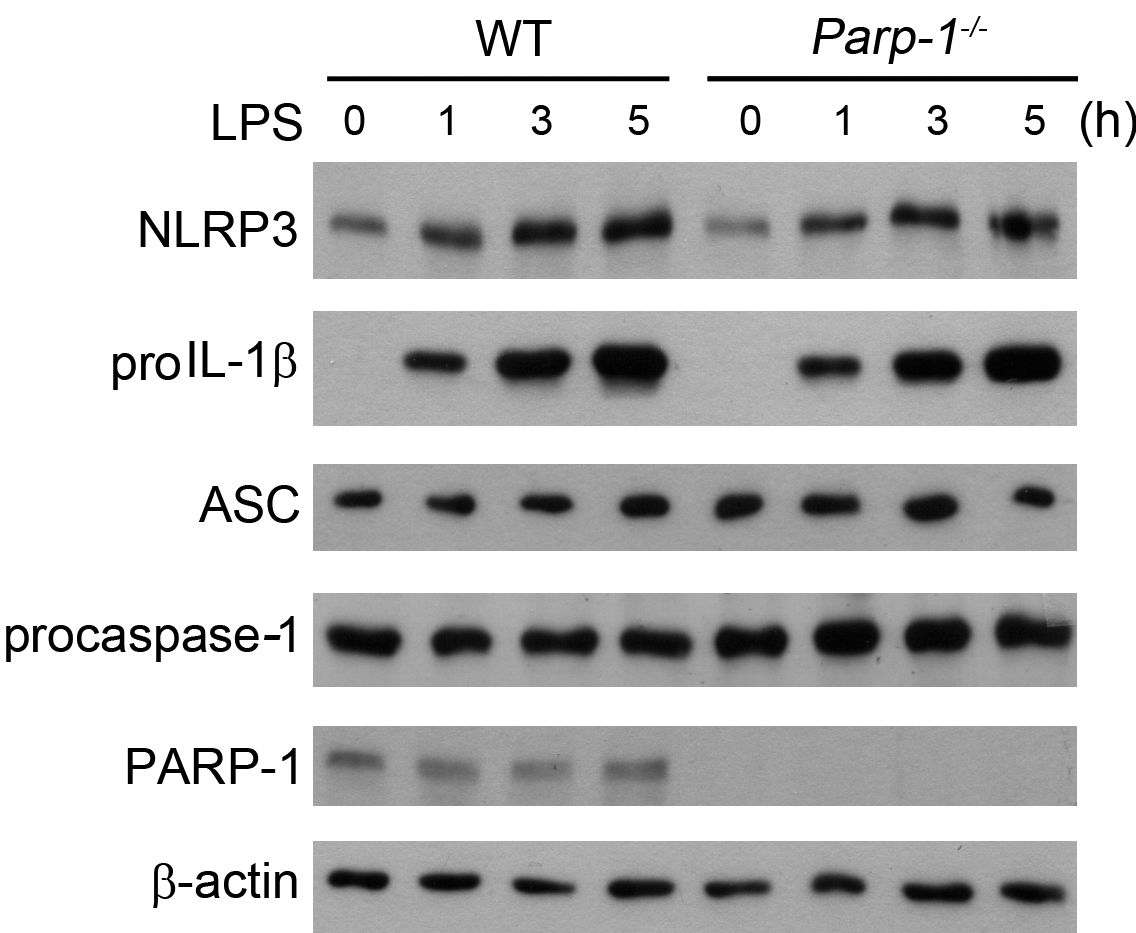

**Figure S2**

(a) Macrophages were treated with LPS (1 μg/mL) for the indicated time and examined for mRNA induction of IL-1β and NLRP3 which normalized to β-actin mRNA and expressed relative to those of untreated control. (b) Macrophages were treated with LPS (1 μg/mL) for the indicated time. Whole-cell lysates were collected for immunoblotting with indicated antibody.
